# Reproductive Success of a Tropical Barn Swallow *Hirundo rustica* Population Is Lower Than That in Temperate Regions

**DOI:** 10.3390/ani13010062

**Published:** 2022-12-23

**Authors:** Li Tian, Yu Liu, Zhuoya Zhou, Huaxiao Zhou, Shengjun Lu, Zhengwang Zhang

**Affiliations:** 1 Key Laboratory for Biodiversity Sciences and Ecological Engineering, Ministry of Education, College of Life Sciences, Beijing Normal University, Beijing 100875, China; 2 Life Science and Technology School, Lingnan Normal University, Zhanjiang 524048, China; 3 Research and Development Center for Watershed Environmental Eco-Engineering, Advanced Institute of Natural Sciences, Beijing Normal University at Zhuhai, Zhuhai 519087, China

**Keywords:** breeding success, tropical zone, temperate zone, temperature, precipitation

## Abstract

**Simple Summary:**

Barn Swallows (*Hirundo rustica*) mainly breed in temperate regions and parts of the tropics. These two regions have remarkably different environments, especially pertaining to their weather conditions. It is interesting to see how the breeding performances of Barn Swallows differ between the two regions and how the weather conditions affect the reproductive success of the Barn Swallow. We monitored the breeding activities of two Barn Swallow populations simultaneously in both tropical and temperate regions in China from 2019 to 2021 and investigated the effects of weather conditions on the reproductive success of the Barn Swallow in the study sites. We found that the reproductive success of the Barn Swallow at the tropical site was lower than that at the temperate site, i.e., swallows at the tropical site laid fewer eggs and had fewer nestlings/successful breeding attempts than swallows at the temperate site. Temperature, rather than rainfall, influenced the breeding performances of Barn Swallows at both sites. In particular, nestling survival decreased with increasing temperatures at the tropical site, but not at the temperate site. We suggest that researchers pay more attention to the reproduction and population changes of the Barn Swallow in the tropics in the context of climate warming.

**Abstract:**

Temperate–tropical comparisons of avian life history traits are helpful to understand the different selective pressures placed on birds by different climate zones. Although there have been many comparative studies targeting multiple species in different regions, there are few comparative studies on the reproductive successes of the same species between tropical and temperate regions. In this study, we monitored the breeding activities of the Barn Swallow (*Hirundo rustica*) simultaneously at a single tropical site and a single temperate site in China, compared the breeding performances of the two populations, and investigated the effects of weather conditions on reproductive success separately. The clutch and brood sizes of the Barn Swallow at the topical site were significantly smaller than those at the temperate site. Furthermore, the breeding success of the Barn Swallow at the tropical site was significantly lower than that at the temperate site. The mean daytime temperature had a negative effect on the clutch size and brood size at both sites; it had a negative effect on nestling survival at the tropical site, but not the temperate site. This study will help us understand the adaptation strategies of widely distributed bird species in different environments, and how climate change will affect birds in different climate zones.

## 1. Introduction

Variations in breeding performances are common phenomena in birds living in different regions. Tropical birds are generally thought to have higher predation pressures, smaller clutch sizes, and lower reproductive success rates than temperate birds [1]. Many life history characteristics (clutch size, egg quality, growth rate, parental care behavior, the age of sexual maturity, and adult survival) often differ significantly between northern and southern bird species [2,3]. For example, a study on the global variations of clutch sizes in 5290 bird species showed the average clutch sizes across assemblages with remarkably strong geographic gradients (from an average of 4.5 eggs in the high northern latitudes to just over 2 eggs in the tropics) [4]. A comparison of temperate and tropical House Wrens (*Troglodytes aedon*) found that tropical wrens had a lower field metabolic rate during reproduction and produced fewer chicks per brood [5]. Although there are several comparative studies on the reproductive outputs conducted on some bird species in different climate zones, only a few studies considered the same species between tropic and temperate regions [6].

Differences in the life history characteristics of birds in different regions may be due to factors such as climatic conditions [7], predation risk [8,9], and food availability [10]. The effect of climate on animal reproduction has been demonstrated by many studies [11,12]. Weather conditions may impose direct selection pressure on bird reproduction; for instance, a study of 327 bird species in Australia found that reproduction may be limited by physiological stress caused by extreme temperatures [13]. Conversely, weather conditions may affect bird reproduction, indirectly by affecting food resources. High temperatures have been shown to reduce the food provisions of the Southern Fiscal (*Lanius collaris*), thereby reducing nestling growth rates and muscle development [14]. Moreover, in different areas, the same climatic factors may have different effects on the reproduction of birds. For instance, precipitation may have a negative effect on food richness in temperate regions, resulting in a remarkable decrease in reproductive success [15]. However, it may have a positive effect on food richness in the tropics, especially in arid regions [16]. While studies exploring how weather conditions affect the reproduction behaviors of the same species in different climate zones are still scarce, such studies help researchers understand the mechanism of animal adaption to climate directly and provide prospects of population trends under global warming [17,18].

The Barn Swallow (*Hirundo rustica*) is an aerial insectivore that breeds widely in a variety of habitats and climate zones across Eurasia and North America [19], making it an ideal model species for studying avian life history strategies. So far, there have been many reports on the reproductive ecology of the Barn Swallow in temperate regions [20,21,22,23,24,25,26,27], but we know little about their reproduction in the tropics (see [28,29]). One preliminary study conducted in subtropical Asia (Nanning, China) found that the Barn Swallow breeds locally from early April to early July, with two to five nestlings per nest. Nestlings in the subtropical area appeared to have lower body mass and longer wings than nestlings from temperate regions [30]. However, as far as we know, the breeding success of the Barn Swallow in tropical regions and corresponding influencing factors have not been reported.

Temperate–tropical comparisons are fundamental to understanding the different selective pressures that birds face in different climate zones and have been applied most frequently to study life history evolution [5,31]. Here, we studied the breeding ecology of the Barn Swallow in tropical and temperate regions simultaneously, compared the reproductive performances of the Barn Swallow in the two climate zones, and explored the potential impacts of weather factors (precipitation and temperature) on the reproductive performances of the Barn Swallow. Considering the temperate–tropical life history differences [1,2,3] and that the abundance of aerial insectivores may be affected by precipitation [19,32], we predicted that (1) the breeding success of the Barn Swallow at the tropical site would be lower than that at the temperate site; and (2) precipitation and temperature would have different effects on the reproduction of the Barn Swallow at the tropical and temperate sites.

## 2. Materials and Methods

### 2.1. Study Sites

**Figure 1:**
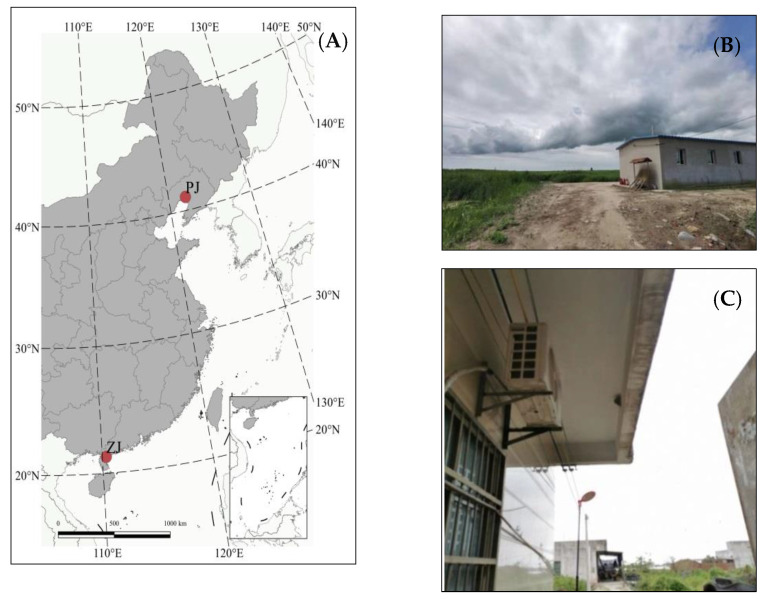
The locations (**A**) and habitats (**B**) Panjin, (**C**) Zhanjiang of the studied tropical and temperate Barn Swallow populations. Panjin (PJ) is in a temperate region and Zhanjiang (ZJ) is in a tropical region.

This study was conducted in Zhanjiang city, Guangdong Province, China, from March to July 2020 and 2021, and in Panjin City, Liaoning Province, China, from May to August 2019 and 2021 (Figure 1). Zhanjiang is the southernmost coastal city in mainland China. Zhanjiang, located in the Leizhou Peninsula, is bordered by the South China Sea to the east and Qiongzhou Strait to the south. The annual average temperature and precipitation are about 23.3 °C and 1640 mm, respectively. This area has a northern tropical monsoon climate, perennially influenced by tropical marine warm and humid air currents. The annual precipitation varies greatly between years, with uneven space–time distribution and obvious seasonal differences [33]. Zhanjiang City has a population of about 7 million and a total land area of 13,225 km^2^, with 6103 km^2^ of arable land, mainly planted with rice, bananas, and sugarcane [34]. Zhanjiang research sites include villages of the Mazhang District (21.2732° N–21.2652° N, 110.1513° E–110.2908° E) and Fucheng Town (20.8963° N–20.8792° N, 110.1513° E–110.1595° E). The studied Barn Swallow population (from 5 villages in Zhanjiang) consisted of about 40–100 breeding pairs for each village each year. The nest sites of the Barn Swallow in the Mazhang District were reinforced concrete buildings with 1 to 8 stories, and the surrounding habitats included grassland and shrubs. The nesting environment in Fucheng Town included buildings similar to those in the Mazhang District and traditional brick buildings, and the surrounding habitat was mainly fish ponds, rice fields, and grassland. The Panjin research site is located in the Liao River Estuary National Nature Reserve (40.9668° N–41.0243° N, 121.6729° E–121.7433° E). It has a warm temperate monsoon climate, with an average annual temperature of 8.3 °C and average annual precipitation of about 600 mm [35]. Panjin City has a population of about 1.3 million and a total land area of 4071 km^2^; the main terrain consists of flood plains and tidal flats [36]. The studied Barn Swallow population in Panjin consisted of about 90 breeding pairs each year; they were mainly bred in traditional brick and tile buildings. Those buildings were distributed in 5 sparsely separated sites, between which, the distances were about 3 km and the surrounding habitat vegetations were mainly reed ponds. The two study sites, Zhanjiang and Panjin, share some similarities; for instance, they are both coastal cities with altitudes at about the sea level; both cities have high air quality and no significant air pollution occurred during the study; no livestock or grazing was found at the study sites. However, considering that Zhanjiang has a higher population density and the study site in Panjin is within a national nature reserve, Zhanjiang has a higher level of urbanization than Panjin.

### 2.2. Nest Monitoring

During the study period, all Barn Swallow nests found at the study sites were numbered and inspected every 3 to 5 days to record the laying dates of the first eggs, clutch sizes, hatching dates, brood sizes at hatching (not recorded in Panjin, 2019), brood sizes of 11/16-day-old nestlings, nest fate, and the cause of breeding failure. GPS data of the nests were recorded. It was recorded as a breeding attempt if newly laid eggs were found in the nest. In Panjin, we defined the fate of nests that still had live nestlings 11 days after hatching as successful breeding attempts because nestlings rarely died afterward before fledging (97.35% of 226 nestlings from 54 broods survived from 11 to 16 days old in 2022; the paired T-test for comparing the mean brood size of 11 and 16-day-old nestlings: T = 1.43, df = 53, *p* = 0.16). In Zhanjiang, due to the high mortality rate of nestlings, we extended the monitoring period and defined the fate of nests with live 16-day-old nestlings as successful. We recorded the causes of breeding failure and classified them into the following categories: (1) nest damage—refers to the suspension of breeding activities due to the damage or fall of nests caused by bad weather or human destruction; (2) hatching failure—refers to the nest beyond the incubation period without chicks; (3) egg disappearance—refers to the disappearance of eggs observed in the nest during the incubation period; (4) nestling disappearance—refers to nestlings that disappeared within 15 days of age; (5) nestling death—refers to the phenomenon of nestling death observed during the brooding period; (6) interspecies competition—refers to the phenomenon where Barn Swallow nests were occupied by other bird species and the reproduction was forced to stop.

### 2.3. Breeding Stage and Meteorological Data

The breeding process of the Barn Swallow could be divided into the following stages: the egg-laying period (from the first egg to the penultimate egg [19]), incubation period (from the day when the last egg is laid to the day before the first nestling hatches), and brooding period (from the day of chick-hatching to the day when the nestlings are 10 days old). We used the meteorological data published by Raspisaniye Pogodi, Ltd. (https://rp5.ru/, accessed on 1 October 2021). The number of Zhanjiang meteorological stations is 59,658, about 15–40 km away from our study sites in Zhanjiang; the number of Panjin meteorological stations is 54,337, about 60 km away from our study site in Panjin. The meteorological data consisted of average temperatures, precipitation, etc., for no longer than every three hours, from which daily rainfall and average temperatures were extracted. For each stage, we calculated the mean daytime temperature and daytime precipitation totals.

### 2.4. Statistical Analyses

Statistical analyses were performed in R software (Microsoft R Open 4.0.2, R Core Team, Vienna, Austria [37]) and RStudio (2022.02.0 + 443, RStudio, PBC, Boston, MA, USA). For each region, we calculated the breeding success, daily nest survival rate, and daily egg survival rate. The breeding success, also known as the apparent survival of nests, was calculated as the ratio between the number of successful breeding attempts and the total number of breeding attempts [38]. RMark (R package RMark 2.2.7, [39]) was used to calculate the daily survival rate (DSR), including the daily nest survival rate and daily egg survival rate [40,41]. Four variables were used to construct the daily survival model: (1) the date of discovering the nest; (2) the last survival date of the nest; (3) the date when the fate of the nest was determined; (4) nest fate (success or failure).

For each nest, we calculated hatching success and nestling survival. The hatching success was calculated by dividing the brood size at hatching by the clutch size. The nestling survival was calculated by dividing the brood size of 11-day-old nestlings by the brood size at hatching.

A chi-square test was used to compare the breeding success of the Barn Swallow populations at the tropical and temperate sites. Since the variances of data were not equal between the two studied populations (Levene’s test: F = 11.06–15.05, df = 1, both *p* < 0.01), Welch’s *t*-tests were used to compare the clutch size and the brood size of 11-day-old nestlings between tropical and temperate populations. Wilcoxon rank-sum tests were used to compare the hatching success and nestling survival because the data were not normally distributed.

Generalized linear mixed models (GLMMs, glmer function, lme4 package) were established to analyze the effects of weather conditions on the breeding performances of the Barn Swallow. GLMMs were established for the two study sites (Zhanjiang, Panjin), respectively. The response variables were clutch size, hatching success, the brood size of 11-day-old nestlings, and nestling survival. The fixed factors were the mean daytime temperature and precipitation of the corresponding reproductive stage (clutch size: egg-laying period; hatching success: incubation period; brood size and nestling survival: brooding period), and the random factor was the year (except for hatching success and nestling survival in Panjin with only one year of data available for the analysis). For the brood and clutch sizes, a normal distribution was selected to describe the error structures of the response variables, while a binomial distribution with a logit link function was used for hatching success and nestling survival. Apart from constructing GLMMs separately for each study site, we also constructed GLMMs with the dataset including both sites and with the site, the interaction between the site, and weather conditions as fixed factors for each response variable.

## 3. Results

### 3.1. Breeding Season, Clutch Size, and Brood Size

**Figure 2:**
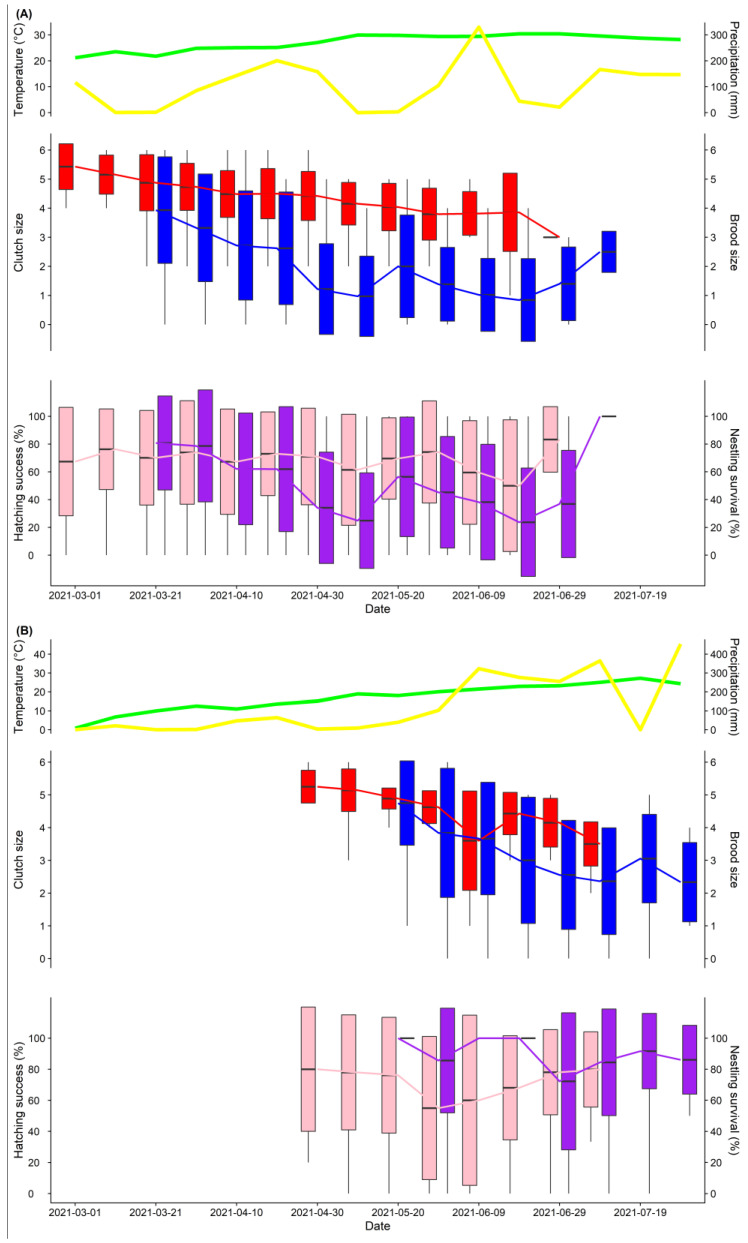
Weather (temperature, precipitation) and the reproductive performances of the Barn Swallow in the two study sites from March to July 2021. Egg-laying dates were from late February to early July at the tropical site (**A**) and from late April to late July at the temperate site (**B**). Upper part: the mean daytime temperature is shown in green and the total daytime precipitation is shown in yellow. Middle and lower part: Clutch size (**red**), brood size (**blue**), hatching success (**pink**), and nestling survival (**purple**) are shown with boxplots, in which the means, mean ± SD, and the range of the data are shown. Data are presented in 10-day periods.

At the tropical site (Zhanjiang), the breeding season of the Barn Swallow started from the end of February to the end of July, lasting about five months in total. At the temperate site (Panjin), the breeding season of the Barn Swallow started from early May to mid-August and lasted about three and a half months (Figure 2). The reproductive parameters of the Barn Swallow in the two sites are shown in Table 1. Both the clutch size and brood size of the Barn Swallow at the tropical site were significantly smaller than at the temperate site (both *p* < 0.001).

### 3.2. Reproductive Success

Hatching success showed no difference between the two Barn Swallow populations at the tropical and temperate sites from 2019 to 2021 (*p* = 0.345). However, the nestling survival of the Barn Swallow at the tropical site was significantly lower than at the temperate site (*p* < 0.001, Table 1).

The breeding success of tropical Barn Swallows was significantly lower than that of temperate swallows (*p* < 0.001, Table 1). The daily egg survival rate of Barn Swallows at the tropical site was the same as that at the temperate site. The daily nest survival rate of tropical Barn Swallows appeared to be lower than that of temperate Barn Swallows. Within a reproductive cycle, nest daily survival appeared to be lower than egg daily survival at the tropical site, whereas it was the opposite at the temperate site (Table 1).

### 3.3. Reproductive Failure

The most important reason for reproductive failure at the temperate site was nest damage caused by rainfall (54.35%), followed by nestling disappearance (15.22%) and egg disappearance (13.04%). At the tropical site, the most important reason for reproductive failure was nestling disappearance (60.58%), followed by egg disappearance (12.50%), hatching failure (12.50%), and nestling death (11.54%). The nestling disappearance of Zhanjiang was significantly higher than that at the temperate site (*X*^2^ = 29.27, df = 1, *p* < 0.001), and the egg disappearance showed no difference between the two Barn Swallow populations at the tropical and temperate sites (*X*^2^ = 9.39 × 10^−31^, df = 1, *p* = 1).

### 3.4. Effects of Weather Conditions on Reproduction

The mean daytime temperature at which Barn Swallows started breeding at the tropical site was above 20 °C, while it was above 16 °C at the temperate site (Figure 2, Figures S1 and S2). At the tropical site (Zhanjiang), the mean daytime temperature during the breeding season of the Barn Swallow (from March to July 2020 and 2021) was 28.09 ± 3.83 °C (mean ± SD, *n* = 304). At the temperate site (Panjin), the mean daytime temperature during the breeding season (from May to July 2019 and 2021) was 19.08 ± 7.72 °C (mean ± SD, *n* = 303). The mean daytime temperature at the tropical site was significantly higher than at the temperate site (T = 18.196, df = 441.62, *p* < 0.01). In terms of precipitation, the distribution of precipitation at the tropical site from March to July was not regular, while precipitation increased after May at the temperate site (Figure 2).

**Figure 3:**
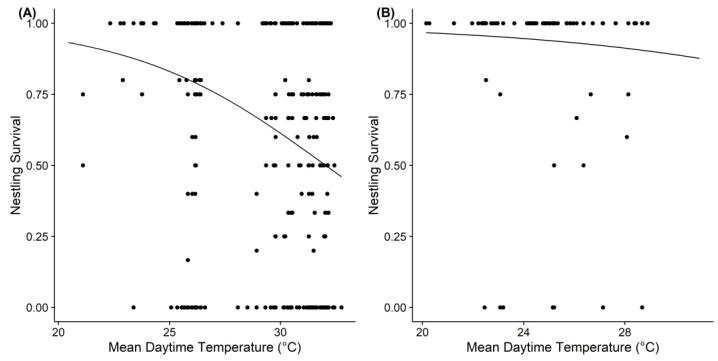
Mean daytime temperature in the brooding period had a negative effect on nestling survival at the tropical site (**A**), but not at the temperate site (**B**). The figure shows the fitted regression lines of GLMMs.

The results of the GLMMs between the reproductive parameters and local weather conditions showed that the temperature affected the clutch size, brood size, and nestling survival of the Barn Swallow, but those effects were not completely the same at the different study sites (Table 2 and Table S1). We found that the mean daytime temperature during the egg-laying or incubation periods had a negative effect on the clutch size or brood size of the Barn Swallow at both sites. The mean daytime temperature during the brooding period also had a negative effect on the nestling survival of swallows at the tropical site, but not on that of temperate swallows (Figure 3). Precipitation had no significant effects on the reproductive parameters of the Barn Swallow at both study sites.

## 4. Discussion

### 4.1. Barn Swallows Had Lower Reproductive Success at the Tropical Site

When comparing the breeding success of tropical and temperate Barn Swallows, we found that the breeding success of Barn Swallows at the tropical site was significantly lower than that of swallows at the temperate site. In tropical Zhanjiang, only 38.00% of breeding attempts had at least one young fledged, while 75.08% of attempts in temperate Panjin had at least one young fledged. Furthermore, the clutch size of Barn Swallows at the tropical site was significantly smaller than at the temperate site, resulting in 1.47 nestlings fewer on average. Considering there was no difference between the hatching success of the Barn Swallow in the tropical and temperate sites, we assumed that the main reason for the lower breeding success at the tropical site was the lower nestling survival in tropical Barn Swallows.

Many studies have found that tropical birds generally have relatively lower breeding success than temperate species. For instance, the breeding success of the White-rumped Munia (*Lonchura striata*) in tropical Yunnan, China, was 31.76% [42]. The Fork-tailed Flycatchers (*Tyrannus savana*) in tropical south-eastern Brazil showed a breeding success of 25% [43]. Comparing our results with the reproductive performance of other Barn Swallow populations or subspecies also support this view. Both Barn Swallow populations in this study belong to the subspecies *H. r. gutturalis* [44], while the temperate (Panjin) population had a similar reproductive performance with other subspecies from temperate regions, the tropical (Zhanjiang) population had lower reproductive success than temperate ones. For instance, the breeding success ranges from 65% to 95.7% in European Barn Swallow populations (*H. r. rustica*) [24,45,46,47,48] and from 78.5% to 92.3% in North American populations (*H. r. erythrogaster*) [19,49], while the figures are 75.08% in Panjin and 38.00% in Zhanjiang, respectively. These results support the hypothesis that the successful rearing of offspring is more challenging in the tropics than in temperate regions.

For the daily survival rate, the daily egg survival rate was the same in the tropical and temperate sites in this study, indicating that the daily survival in both sites was similar during the egg stage, which was also supported by the similar hatching success. The daily nest survival rate appeared to be higher than egg daily survival at the temperate site. Studies on the nest survival of the Laughing Dove (*Spilopelia senegalensis*) have shown that the daily nest survival rate increased with the increasing nest age, possibly due to the increased intensity of parental defensive behavior with reproduction progress [50]. The different patterns at the tropical site, i.e., a higher daily egg survival rate and a lower daily nest survival rate may be related to greater nest predation pressure [51].

### 4.2. Reproductive Failure

Overall, the reasons for reproductive failure in this study are similar to those in other Barn Swallow studies, including nest damage, hatching failure, and egg or nestling disappearance [24,27,52,53]. However, the challenges that Barn Swallows faced in breeding were different in temperate and tropical regions. The main reason for breeding failure at the temperate site was wet conditions and the aging or dilapidation of the buildings used by the Barn Swallow, leading to nests dropping from the buildings. At the tropical site, the main cause of reproductive failure was the disappearance of eggs and the death and disappearance of nestlings. Nestlings may disappear due to (1) predation; (2) infanticide caused by intraspecific competition; and (3) nestlings falling out of their nests when begging for food (L.T. and Y.L., personal observation). A similar study on an Argentina Barn Swallow population found that 57% of eggs did not produce fledglings. These failures were due to predation (29%), ectoparasites (15%), and undefined causes (56%) [26]. It is concerning that we have detected many cases of nestlings dying in the nest, probably due to the weather or a mismatch between parental provisioning and the need for nestlings [54]. Therefore, we recommend that monitoring devices be installed to further explore the causes of egg disappearance, nestling disappearance, and nestling death in tropical regions.

### 4.3. Weather Factors

Studies have shown that weather conditions can directly [55,56] or indirectly [55,57] cause changes in bird reproductive performances and offspring quality. For instance, some South American Barn Swallow nestlings leave the nest along the nesting substrate before their first flight, which often occurs during periods of extreme heat when nestlings need to seek cooler locations even when they are 12 days old [26]. Conversely, food availability for Barn Swallows may be affected by weather conditions. In a comparative study of insect abundance and the breeding success of the Barn Swallow in two urban habitats, it was found that food shortages caused by severe weather led to high mortality in nestlings under 12 days of age [49]. In the present study, we found that the temperature was a limiting factor for reproduction at the tropical site, whereas temperature affected the clutch size and brood size at the temperate site, but did not affect nestling survival. While global warming influences organisms in the tropics even more strongly [58], the mechanisms by which high temperatures in tropical areas reduce the nestling survival of Barn Swallows remain to be explored. 

Contrary to our expectations, precipitation does not affect the nestling survival of the Barn Swallow at either temperate or tropical sites. It was reported that the average mass of insects brought to swallow nestlings was positively related to the temperature and negatively related to rainfall [15]. Another study conducted in the tropical region of Xishuangbanna, China, also showed that rainfall and temperature had important effects on the reproductive phenology and reproductive performance of the White-rumped Munia [16]. Considering that Barn Swallows feed on flying insects, continuous precipitation may affect the density of insects in the air and lead to a decrease in food resource richness. Thus, further research needs to be conducted on how Barn Swallows adjust their parental investments to compromise food shortages caused by rain, or whether the nestling body condition rather than survival decreases with precipitation in the studied populations, especially considering that global warming will exacerbate extreme weather events [17,18].

### 4.4. Weakness of the Study

In this study, we focused on the reproductive performances of Barn Swallows in different climate zones and the influence of weather factors on reproductive performances. However, many other factors can also affect the reproductive performances of Barn Swallows, such as habitat [59], parasites [60], nest sites [23,61], urbanization [62,63], etc. Although our studied sites shared some similarities in environmental factors, the observed differences in reproductive performances between the sites could still be affected by other factors other than weather conditions. To exclude the potential effects of other factors on the reproduction of the Barn Swallow, we plan to collect more breeding data from more populations, especially tropical populations, to verify the effects of weather conditions. In addition, since it is hard to directly observe the reasons for reproductive failures, such as nest predation, we attempted to install infrared cameras for recording, but the effectiveness was not ideal. Hopefully, we will improve the technique and equipment for monitoring the reproduction of the Barn Swallow and identify the causes of egg and nestling disappearances in future research.

## 5. Conclusions

We provided the first report on the breeding success of the Barn Swallow in the tropics. By comparing the reproductive performances of tropical and temperate Barn Swallows, we found that the reproductive success of Barn Swallows at the tropical site was lower than that at the temperate site. Temperature, rather than precipitation, had a strong effect on the breeding performance of the Barn Swallow. The nestling survival decreased with increasing temperatures at the tropical site, but not at the temperate site. Further identifying the effects of weather conditions on the reproduction of the Barn Swallow, especially in the tropics, requires collecting data from more populations. We suggest that more attention be given to the reproductive success and population changes of the Barn Swallow in the tropics in the context of global warming.

## Figures and Tables

**Table 1:** Reproductive parameters of two Barn Swallow populations in tropical (Zhanjiang) and temperate (Panjin) climate zones.

	Zhanjiang	Panjin	T/Wilcoxon Rank-Sum/Chi-Square Test
Breeding success	38.00% (*n* = 729)	75.08% (*n* = 305)	*X*^2^ = 39.80, df = 1, *p* < 0.001
Clutch size	4.33 ± 0.87 [4.27, 4.38] (*n* = 854)	4.62 ± 0.80 [4.53, 4.71] (*n* = 299)	T = −5.29, df = 564.39, *p* < 0.001
Brood size	2.14 ± 1.71 [1.99, 2.29] (*n* = 504)	3.61 ± 1.54 [3.41, 3.80] (*n* = 253)	T = −11.75, df = 539.58, *p* < 0.001
Hatching success	0.73 ± 0.31 [0.71, 0.76] (*n* = 590)	0.73 ± 0.36 [0.66, 0.79] (*n* = 118)	W = 32,977, *p* = 0.345
Nestling survival	0.54 ± 0.43 [0.51, 0.59] (*n* = 411)	0.89 ± 0.28 [0.84, 0.95] (*n* = 99)	W = 10,836, *p* < 0.001
DSR egg	98.97% (*n* = 854)	98.97% (*n* = 299)	
DSR nest	97.76% (*n* = 729)	99.05% (*n* = 229)	

DSR egg: daily egg survival rate; DSR nest: daily nest survival rate. Means are presented with standard deviation and 95% confidence intervals.

**Table 2:** Effects of weather conditions on the reproduction of the Barn Swallow. Estimates, standard error, T/Z values, sample size, and *p* values of the GLMMs are presented. For each response variable, weather conditions (temperature, precipitation) of the corresponding breeding period were analyzed (clutch size: egg-laying period; hatching success: incubation period; brood size and nestling survival: brooding period). Significant differences (*p* < 0.05) are indicated by bolded font.

	Zhanjiang					Panjin				
	Estimate	SE	T/Z	*n*	*p*	Estimate	SE	T/Z	*n*	*p*
**Clutch size**										
Intercept	6.208	0.316	19.619	632	**<0.001**	7.242	0.449	16.144	252	**<0.001**
Temperature	−0.069	0.010	−6.972	632	**<0.001**	−0.110	0.018	−6.072	252	**<0.001**
Precipitation	−4.472 × 10^−5^	0.001	−0.046	632	0.964	−0.001	0.002	−0.756	252	0.450
**Hatching success**										
Intercept	3.492	1.286	2.716	523	**0.007**	4.944	2.805	1.763	99	0.078
Temperature	−0.060	0.044	−1.358	523	0.174	−0.129	0.117	−1.103	99	0.270
Precipitation	0.001	0.001	0.601	523	0.548	−0.001	0.004	−0.187	99	0.852
**Brood size**										
Intercept	7.311	0.786	9.303	504	**<0.001**	7.735	0.982	7.876	245	**<0.001**
Temperature	−0.172	0.025	−6.863	504	**<0.001**	−0.155	0.035	−4.387	245	**<0.001**
Precipitation	−0.001	0.001	−1.199	504	0.231	−0.002	0.002	−1.089	245	0.279
**Nestling survival**										
Intercept	7.279	1.340	5.432	411	**<0.001**	6.025	4.136	1.457	98	0.145
Temperature	−0.226	0.042	−5.365	411	**<0.001**	−0.131	0.158	−0.830	98	0.406
Precipitation	<0.001	0.001	−0.341	411	0.733	−0.005	0.005	−0.993	98	0.321

## Data Availability

The data presented in this study are available upon request from the corresponding author.

## Supplementary Materials

The following supporting information can be downloaded at: https://www.mdpi.com/article/10.3390/ani13010062/s1, Figure S1: Weather (temperature, precipitation) and reproductive performances of Barn Swallows in Panjin from March to July 2019; Figure S2: Weather (temperature, precipitation) and reproductive performances of Barn Swallows in Zhanjiang from March to July 2020; Table S1: Effects of study sites, weather conditions, and their interactions with the reproduction of the Barn Swallow.
