# Supplementary material for: Reproductive Success of a Tropical Barn Swallow Hirundo rustica Population Is Lower Than That in Temperate Regions
Source: Animals (Basel). 2022 Dec 23;13(1):62. doi: 10.3390/ani13010062 (PMC9817904; doi:10.3390/ani13010062)
Supplement: Supplementary file 1 [file animals-13-00062-s001.zip › animals-2059658-supplementary.pdf]

animals MDPI

Supplementary Materials

Table S1. Effects of study sites, weather conditions and their interaction on the reproduction of the Barn Swallow. Estimates, standard error, T/Z values, sample sizes and P values of the GLMMs are presented. For each response variable, weather conditions (temperature, precipitation) of corresponding breeding period were analysed (clutch size: egg laying period; hatching success: incubation period; brood size and nestling survival: brooding period). Significant differences ($p < 0.05$) are indicated by bolded font.

	Estimate	SE	T/Z	*n*	*p*
**Clutch size**					
Intercept	7.095	0.493	14.380	884	**<0.001**
site	-0.754	0.534	-1.411	884	0.159
temperature	-0.109	0.020	-5.547	884	**<0.001**
precipitation	-0.002	0.002	-0.704	884	0.482
site * temperature	0.041	0.022	1.860	884	0.063
site * precipitation	0.001	0.002	0.624	884	0.533
**Hatching success**					
Intercept	4.944	2.804	1.763	622	0.078
site	-2.656	3.010	-0.882	622	0.378
temperature	-0.129	0.117	-1.103	622	0.270
precipitation	-0.001	0.004	-0.187	622	0.852
site * temperature	0.098	0.123	0.793	622	0.428
site * precipitation	0.002	0.004	0.347	622	0.729
**Brood size**					
Intercept	7.817	1.033	7.569	749	**<0.001**
site	-0.417	1.248	-0.334	749	0.739
temperature	-0.155	0.038	-4.034	749	**<0.001**
precipitation	-0.002	0.002	-1.112	749	0.269
site * temperature	-0.017	0.045	-0.377	749	0.706
site * precipitation	0.001	0.002	0.428	749	0.670
**Nestling survival**					
Intercept	6.045	4.264	1.418	509	0.156
site	1.233	4.423	0.279	509	0.780
temperature	-0.107	0.163	-0.658	509	0.510
precipitation	-0.004	0.005	-0.913	509	0.361
site * temperature	-0.119	0.168	-0.707	509	0.480
site * precipitation	0.004	0.005	0.785	509	0.433

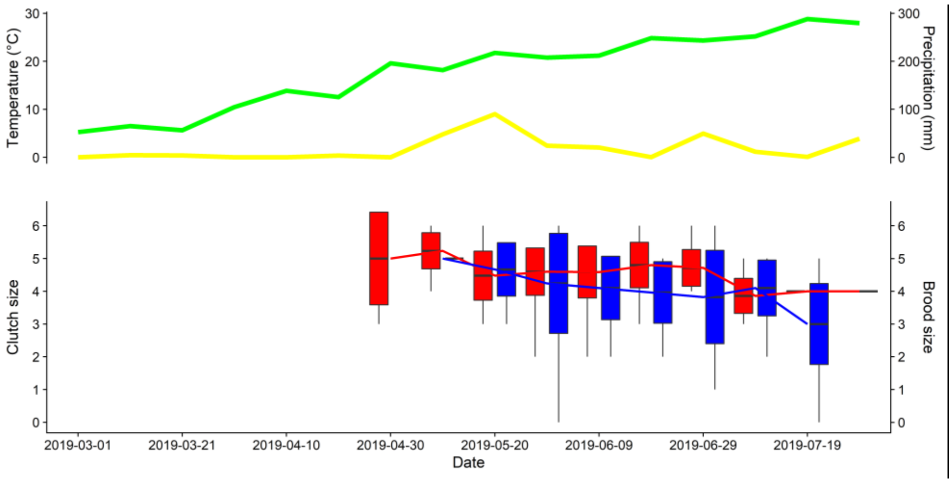

Figure S1. Weather (temperature, precipitation) and reproductive performance of the Barn Swallow in Panjin from March to July, 2019. Upper part: the mean daytime temperature is shown in **green** and total daytime precipitation is shown in **yellow**. Lower part: Clutch size (**red**) and brood size (**blue**) are shown with boxplots, in which means, mean ± SD, and the range of the data are shown. Data are presented in 10-day-periods.

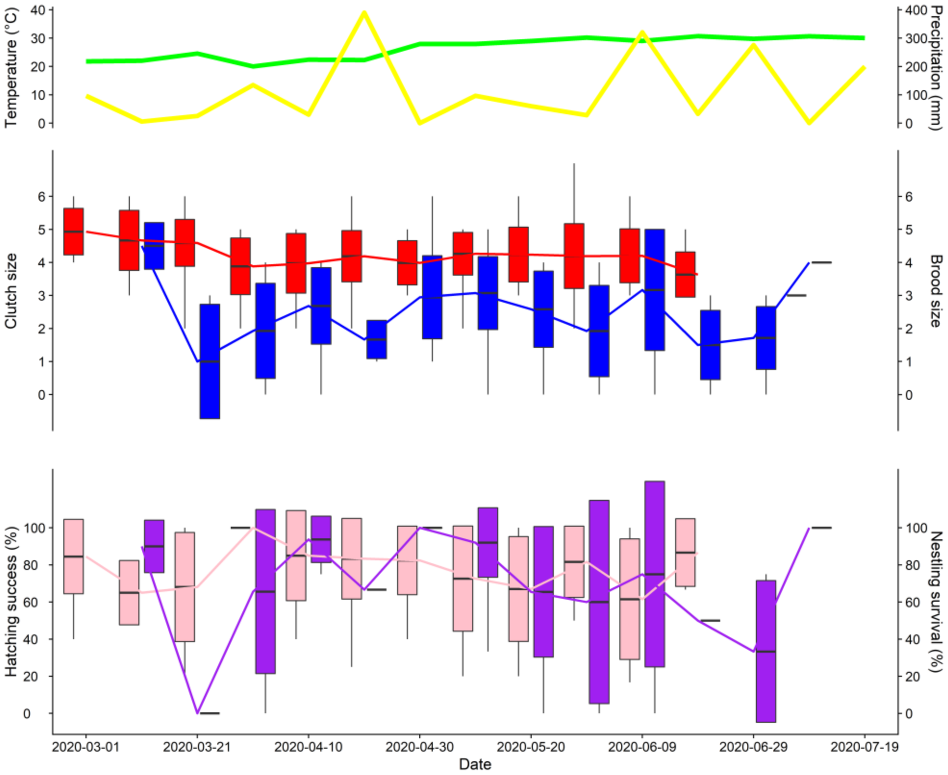

Figure S2. Weather (temperature, precipitation) and reproductive performance of the Barn Swallow in Zhanjiang from March to July, 2020. Upper part: the mean daytime temperature is shown in **green** and total daytime precipitation is shown in **yellow**. Lower part: Clutch size (**red**), brood size (**blue**), hatching success (**pink**) and nestling survival (**purple**) are shown with boxplots, in which means, mean ± SD, and the range of the data are shown. Data are presented in 10-day-periods.
